# Selections and Abstracts

**Published:** 1902-10

**Authors:** 


### SELECTIONS AND ABSTRACTS.

---

### How to Assist Young Girls to Womanhood.

BY EDWARD C. HILL, M.D., DENVER, COLORADO.

The primary establishment and the menopausal cessation of menstruation are the two crucial physical epochs of woman's life. The change from maidenhood to womanhood is one that involves the whole body, and manifests itself alike in the form, the voice and the sexual and nervous phenomena. In an ideal state of perfect health this transition into puberty should be as natural and uneventful as gliding from sleep into consciousness. Owing, however, to the present civilized modes of living, the cerebral development of young girls is fostered and forced to a degree that deprives the remaining tissues and organs of their necessary nutrition, and too often we are called upon to treat delicate girls that are like buds blasted in the blossoming. Many a woman traces back a prolonged existence of semi-invalidism to exposure and lack of care at the early menstrual periods. Tight lacing also predisposes to pelvic disorders by interfering with circulation and exciting uterine displacements. The strain of puberty upon the nervous and blood-forming structures may be too great in a subject hereditarily deficient in vital resistance and adaptability. So we may count among the morbid incidents more or less peculiar to puberty, chlorosis and anemias, general debility, neurasthenia and hysteria, acute pneumonic phthisis, chorea and hebephrenia.

According to Emmet, more than half of all women who have suffered at puberty from menstrual derangements are sterile and delicate in after life. Skene has stated that his observations showed that the vast majority of incurable diseases peculiar to women originate in imperfect development and consequent derangement of function. This development is either primary, during the embryonic stage, or secondary, at puberty. Defects in the former are irremediable, whereas secondary deviations from the normal standard are both preventable and curable in most instances.

It is important in connection with the subject under consideration to bear in mind the essential reciprocal relations of the reproductive system and the general organization. As Virchow says, all the specific properties of woman's body and all her womanly characteristics depend upon her ovaries. In other words a woman is not fully a woman unless her sexual development is natural and complete and in line with a healthy general organization. A beautiful illustration of sexual dimorphism has been furnished by Prof. Max Weber (quoted by Skene), who presented the case of a chaffinch in which the left side of the body had the female coloration and the right side that of the male bird, the two colors being sharply limited at the middle line. The bird was a hermaphrodite with a well-developed ovary on the side of the female plumage, and a testicle on the opposite side. The phenomena of menstruation offer the most palpable evidence of the onset of puberty. The precise nature of this rhythmic cycle is overshadowed by a jungle of theories, and, as Millikin well says, we can do no better in the present state of our knowledge than accept menstruation as a habit which has been nailed upon our race by heredity, and which is for us an ultimate biologic fact.

Normal menstruation in temperate climates generally begins in the fifteenth year. In the tropics it appears much earlier, so that in Mexico one may see a grandmother of only twenty years. Within the Arctic Circle Eskimo girls do not generally arrive at puberty until the eighteenth year. City girls usually have the menstrual flow earlier than do hard-working country girls, in whom muscular exercise has the same derivative effect on the pelvic blood supply as too intense devotion to study. The time, amount and character of the menstrual flow vary normally within wide limits. The menstrual cycle for different individuals ranges in perfect health from two to six weeks. The average duration in the temperate zone is about four days. Soaking more than three napkins daily is considered abnormal. Anemic girls, as a rule, tend to menorrhagia; chlorotic ones, to scanty menstruation. Clots are present when the amount of blood is great, or the mucus and fatty acids scanty. A periodic white menstruation, from supersecretion of the uterine glands, is not infrequently noticed in the intervals midway of menstruation.

Menstruation is or should be a perfectly physiologic process. In the virgin disorders of menstruation of whatever nature are nearly always dependent upon the defective nutrition of the reproductive organs, and this in turn upon a blood supply insufficient in quality or in quantity. In the great majority of cases, therefore, our efforts to aid nature in effecting the transformation of the girl into a woman, should be in the line of a happy balance of nutrition between the special female organs and the body as a whole.

Hygienic measures are of the first importance. Fresh air and sunshine are always in order. Exercise is especially indicated for the fat and flabby chlorotic girl, and her diet should be restricted in sugars and starches. The highly active, intellectual girl must rest from her studies and try to become a little lazy. Proper precautions should be taken in regard to reasonable care of the person at the time of the monthly periods. Yet the physician should beware of unduly alarming his little patient, and so bringing about a condition of hypochondriacal valetudinarianism. Simple cleanliness is certain to do no harm, but good. The conservation of the general health and vigor is the chief factor in maintaining safe and easy menstruation.

In spite of hereditary defects, if the physician could have full control of the diet, clothing, hygiene and environments of the little girls in his clientele up to the date of puberty, but little if any medication would be then required. Unfortunately, however, the lack of harmonious development in the preadolescent period necessitates considerable medical attention to secure a normal course for the critical metamorphosis of puberty, whose influences, as Dudley remarks, are fundamental, not only in the reproductive organs, but in the entire woman. Actual pain at the menstrual period in the young virgin may be considered always pathologic, and the same is true of menorrhagia or very scanty menstruation. Such abnormalities of function should direct our attention to the state of nutrition especially. The obese, chlorotic girl must take more exercise; the thin, delicate, sensitive girl, more rest. Fresh air and sunshine are needed in every instance. Red meat, eggs and other bloodforming foods should be taken in such quantities as can be well borne. The appetite for wholesome nutriment should be encouraged, if need be, by stomachic stimulants, such as the official elixir of strychnin, pepsin and bismuth. The use of bromides, coal-tar analgesics and diffusible stimulants at the menstrual periods can be regarded only as a temporary makeshift.

The most constant and positive clinical sign of imperfect puberty is deficiency of the blood in red corpuscles and hemoglobin, the chlorotic type being perhaps more common than the simple anemic in relation to menstrual disorders. Hemic defects and malnutrition act reciprocally as cause and effect. The oxidizing life of the blood is in the iron it contains, with about one-twentieth as much manganese. The total iron of the adult body amounts to but 2.5 or 3.5 grams, chiefly in the form of hemoglobin. The normal daily content of iron in the food of an average diet is, according to Stockman, from five to ten milligrams. When absorbed, as in health, this food-iron replaces the metal continually lost by disintegration of blood-corpuscles and excretion. The round of iron in the body seems to be from the duodenum to the mesenteric glands, thence to the thoracic duct, the general blood current and the spleen, from where it passes to the liver to be synthetized into hemoglobin for the red cells, on the breaking-down of which the dissociated iron is eliminated by way of the large intestine.

The use of iron in anemic and chlorotic conditions is, of course, a cardinal principle in therapeutics. In girls becoming women to supply a deficiency of erythrocytes or hemoglobin, one might infer at first thought that the best method would be to administer hemoglobin, that is, blood in some form. Chemistry proves, however, that when hemoglobin is taken into the stomach it is changed by the acid there to hematin (causing the coffee-ground color of small gastric hemorrhages), which, according to Cloetta, passes down the alimentary tract without being absorbed.

Most authorities conclude that inorganic compounds of iron in order to be absorbed must first be changed to albuminates by combining with food matters. All albuminous substances are hydrolyzed to peptons before they are capable of absorption. Hence it follows that a peptonate of iron is the preparation most likely to be readily and completely absorbed and assimilated. The best remedy of this composition, I think, is Gude's Pepto-Mangan, which I have used for the past ten years with great satisfaction, particularly in the hemic and nutritive disorders of female puberty.

This neutral solution contains three grains of iron and one grain of manganese in each tablespoonful. The latter ingredient is doubtless to be credited with a large part of the nearly specific effect of the remedy in functional menstrual derangements. The preparation is pleasant to the eye, agreeable to the palate and has the great advantage over inorganic iron compounds of not corroding the teeth, deranging digestion or inducing constipation. According to the nature and severity of the case, the dose varies from a teaspoonful to a tablespoonful. It is well taken in milk or sherry just after meals.

The following brief clinical notes may serve to illustrate the facts above stated. The blood count in each instance was made with the Thoma-Zeiss hemacytometer; hemoglobin was calculated by the Hammerschlag specific gravity method. I need hardly remark that the blood findings at the altitude of Denver are normally higher than at points near sea level.

CASE 1. Jose K., fifteen years, thin, delicate and somewhat strumous, had menstruated irregularly and intermittently for sixteen months; erythrocytes 3,600,000, hemoglobin 58 per cent. She was taken out of school, put on a diet largely protein, given aloin, strychnin and belladonna pills for her bowels, and for her blood, Pepto-Mangan (Gude), a dessertspoonful four times daily after eating. Under this treatment she made an average weekly gain of 1¼ pounds in weight, about 150,000 red cells and 3⅓ per cent. hemoglobin, and was discharged cured in ten weeks.

CASE 2. Alice R., eighteen years, rather stout but pale, with greenish tinge; complained of palpitation and breathlessness on slight exertion; menstruation barely begun and scanty. She was made to take gradually increasing exercise on her bicycle, a cool bath every morning, less carbohydrates and more proteins in her diet, and Pepto-Mangan (Gude) in the dose above mentioned. She recovered from all her morbid symptoms within four months, and has since married and given birth to two healthy children.

CASE 3. Amelia B., twenty-three years old, an overworked servant girl, had suffered since the periods first began, nine year before, with marked dysmenorrhea, the flow being prolonged but rather scanty. The red blood-cells numbered 3,800,000 per cu. m. m., with proportionate oligochromia. She was induced to rest at home and take six eggs daily, along with other nourishing food and Pepto-Mangan (Gude), a dessertspoonful four times daily an hour after food. She made a very rapid recovery, the red cells running up to 4,900,000 within two months and the menstrual periods becoming quite normal. By exercising proper care she has remained well for the past eight years.

CASE 4. Olive M., thirteen years, blonde, thin, active, sensitive, a hard student, just beginning to menstruate, the flow being scanty and accompanied with pain. The blood count was 63 per cent. of normal, the color index 57 per cent. Under treatment similar to that mentioned in the first case, she became round and rosy, menstruated freely and easily, took on 17 pounds in weight and raised the blood findings above the normal at sea level, all within eight months.

CASE 5. Fannie R., seventeen years, active, ambitious, intelligent, had such excruciating pain all through her menstrual periods for two years as to cause actual wasting. Physical examination revealed nothing abnormal except an undersized uterus. She was given Pepto-Mangan in tablespoonful doses three times a day, and was told to lie with the head lower than the hips. After three months' treatment the periods became quite painless, and have remained so for five years.

CASE 6. Flora J., sixteen years old, began to menstruate profusely a year before, since which time she has been always ailing; erythrocytes 3,100,000, hemoglobin 63 per cent. She was given cool baths and massage, a bitter tonic, laxatives and Gude's Pepto-Mangan in dessertspoonful doses. When discharged cured, five months later, the blood count was 4,700,000, hemoglobin 95 per cent.

CASE 7. Maggie W., aged fifteen, clerk in a department store, was extremely chlorotic (hemoglobin 28 per cent.), with a soft, systolic basic murmur and some symptoms of gastric ulcer; menstrual molimina but no flow. She was kept in bed at home, fed largely on meat, fish and eggs, and was given Pepto-Mangan (Gude) thrice daily a tablespoonful at a time. The functional murmur soon disappeared, the iron in the blood came gradually up to normal, the patient lost in weight as she gained in health, and menstruation appeared regularly.

CASE 8. Nora R., fourteen years, healthy in appearance but neurasthenic; no trouble with menstruation, except at this time she became more nervous and developed a rapid pulse and some swelling of the thyroid gland. For this incipient exophthalmic goiter she was kept in bed with a cold pack over the thyroid at the menstrual period, and was given Pepto-Mangan (Gude) steadily for six months in dessertspoonful doses. She has been quite well and free from the symptoms mentioned for over a year.

In conclusion the writer would like to emphasize the peculiar physiologic efficacy of Pepto-Mangan (Gude) in aiding young girls to a normal womanhood, when the crisis of puberty is complicated with any defect in blood-making and nutrition. Its action is prompt and pleasant, and the clinical benefits derived from its use are readily apparent to all concerned. In curable cases it is as nearly specific as any combination of drugs could be.

---

### MEDICAL TREATMENT OF CHOLELITHIASIS.*

Cholelithiasis embraces the formation and presence of biliary concretions. Its medicinal treatment is based upon the rationale of its etiology and pathology. Gall-stones are chiefly composed of cholesterine. Cholesterine is a product of retrograde metamorphosis, which is held in solution in the tissues by lecithin. Its solution in the circulating fluids of the body is maintained by the alkaline salts and the compounds of potassium and sodium with the fatty acids (Lyman).

So long as the bile remains alkaline the cholesterine is held in solution. If calcium be added to bile it unites with the fatty and biliary acids to form insoluble salts no longer capable of holding cholesterine in solution. An excess of organic acids in the liquids

*Read by R. Alexander Bate, A.B., M.D., before the Kentucky State Medical Society, Paducah, Ky., May 7-9, 1902.

and solids of the body cause a liberation of basic calcium from the anatomical structures in which this substance is contained.

Calcium thus put into circulation precipitates cholesterine from the bile. This excess of organic acids in the system is the so-called "arthritic diathesis" or the "acid dyscrasia" described by the French writers. Consequently, biliary calculi are most common after the age of thirty-five, that is, after changes dependent upon the diathesis have begun.

Females are affected three times as often as males. Naunyn states that 25 per cent. of all women have cholelithiasis, and that 90 per cent. of these have borne children. Pregnancy, menstruation, tight lacing, sedentary habits, and the increased frequency of osteomalacia among females render them more liable to the disease. Pregnancy and menstruation favor the increase of organic acids in the system. Tight lacing causes stagnation of the bile. Sedentary habits lessen oxidation and in osteomalacia calcium salts are put into the circulation. Cancer of the gall-bladder, typhoid fever, appendicitis, and disorders attended with intestinal sepsis are among the frequent causes of biliary calculi. Colloid material, the products of inflammation, and bacteria present in the bile not only favor chemical changes but furnish a nucleus for the deposit of cholesterine.

Recently it has been shown that the bile is so feebly antiseptic that bacteria may live in the gall-bladder a considerable length of time. Gall-stones vary in number from one to eight thousand, and they vary in size from a pin-head to a goose-egg. Their formation may take place in the hepatic radicals or in the gall-bladder. Symptoms result only from irritation or obstruction by the calculi. The greatest pain is supposed to result from the spasmodic contraction of the muscular structures upon the stone as it passes through the cystic duct. The obstructing stone usually lies at the termination of the common duct, within the diverticulum of Vater.

The formation of a calculus is likely to be followed by the formation of other calculi, either immediately or at subsequent periods. Thus we find the indications for the medicinal treatment of cholelithiasis are: First, the prevention of the formation of calculi; second, the control of the symptoms and the expulsion of the calculi when formed.

Prophylaxis is applicable to those predisposed but in whom no calculi have been formed, and to those from whom calculi have been removed either by surgical or medicinal means. Prophylactic treatment, then, embraces the treatment of the acid dyscrasia, or the prevention of an excess of organic acids in the system; care of the menstruating and pregnant female, especially after the thirty-fifth year; the removal of all things tending to produce stagnation of the bile; the use of all means to promote its fluidity and outflow; the use of agents promoting biliary and intestinal antisepsis, and the use of lecithin or other solvents of cholesterine in those from whom gall-stones have been removed.

An excess of organic acids in the system must be prevented by increasing oxidation in every way. Out-of-door exercise, bathing, massage, etc., and by a diet requiring as little oxygen as possible and free of calcium salts. For this reason no red meats, tea, coffee, chocolate, tomatoes, strawberries, bananas, nor limestone nor carbonated water should be taken. But the lentils should especially be used, because they not only require less oxygen for their assimilation but even carry oxygen with them. The oxygenated waters, instead of those charged with carbonic acid gas, should be used.

The salicylates promote the fluidity of the bile and together with the terebinthina group perhaps constitute the safest antiseptic. Probably the best antiseptic measures in the gall-bladder are those that prevent biliary stasis and promote normal gall-bladder activity. Local massage is to be especially recommended.

In typhoid fever, appendicitis, and other disorders attended with intestinal sepsis, the terebinthina group perhaps furnish the best prophylactic. They lessen both biliary and intestinal sepsis, besides exercising a somewhat solvent action upon biliary concretions. The treatment of the attack embraces measures for the relief of pain as well as those that facilitate the expulsion of gall-stones.

It is estimated that less than fifty per cent. of individuals affected with gall-stones ever have clinical manifestations. Gall-stone colic is generally conceded to be due to the contraction of the muscular structure upon a calculus as it is forced into the cystic duct, so that in all instances of cholelithiasis attended with pain we are reasonably sure the offending calculus is small enough to have become engaged in the duct itself. Therefore we have positive indications for medicinal treatment.

The first steps in the treatment of the attack must of course be for the control of pain. Anodynes and anesthetics have been used. Coal-tar derivatives, opium, alkaloids, ether, and chloroform have served. Perhaps the speediest relief, with the least undesirable after-effect, is obtained by the hypodermatic use of heroin hydrochloride in combination with atropine. Heroin is more analgesic and less constipating than morphine. Just as when morphine is used, small doses should be frequently repeated, lest the stone pass suddenly and a narcotic rather than an analgesic effect result. The agents assisting expulsion of the calculi may be divided into different classes. The action of one class is due to influence upon the tissues of the biliary tract. The action of the other class is due to influence upon the biliary concretion itself. Those that act upon the tissues perhaps exert a local sedative action, lessening muscular rigidity yet toning up muscular force.

A result is obtained analogous to that of cocain upon the urethra. A spasmodically contracted urethra may resist the passage of the smallest metallic sound when cold, yet if cocain, heat, or other local sedative be applied, a sound of average size may easily pass. The drugs belonging to the first-class are dioscorea villosa, carduus marianus, chionanthus virginica, and probably most cholagogues. The drugs belonging to the second-class exercise a solvent effect upon the different component parts of the calculi, and though a solution may not be effected, assist in the reduction and molding of the calculi. The drugs representing this second-class are lecithin, olive oil, glycerine, the salicylates, ether, chloroform, turpentine, animal soap, nitro-muriatic acid, the succinate of the peroxide of iron, valerianate of amyl, toluylendiamin, pichi, Carlsbad salts, and the alkalies in general.

Lecithin is perhaps the most active solvent of cholesterine known. The action of the various oils is dependent upon the lecithin they contain. At present olive oil affords the chief source for its administration. However, a large manufacturing establishment, in reply to a letter, has informed me that they hope soon to place pure lecithin upon the market. Gall-stones composed of cholesterine, when raised to the temperature of the body, may often be molded as putty into any form, according to pressure. If lecithin does not put the cholesterine of the gall-stone into solution it may so soften it as to permit this molding. The succinate of the peroxide of iron, hydrated, contains a large proportion of nascent oxygen, so that it is useful both as a prophylactic and an assistant in expelling calculi.

Pichi dissolves the mucus and products of inflammation that bind together the cholesterine and calcareous matter. Thus we see medicinal treatment instituted for the removal of the calculi should embrace the dietetic and hygienic measures suggested under prophylactic treatment, together with the combined use of both classes of drugs suggested as facilitating the explusion of biliary concretions. In addition to the lentils and oxygenated waters mentioned, eggs, milk (especially buttermilk), whole wheat and corn breads complete the requirements of a diet list. They furnish oxygen, phosphates and lecithin.

Such local measures as massage, counter-irritants, especially the application of the salicylate of methyl and the use of either hot or cold enemata, are of some service. The internal medication should embrace drugs that favorably modify biliary secretion, that relax the spasmodically contracted ducts, lend tone to normal muscular contractions and assist in molding calculi when possible, added to drugs possessing a solvent action upon the calculi. Therefore, the ideal prescription would contain lecithin as solvent for the cholesterine, pichi to dissolve the matrix of mucus, and dioscorea to assist expulsion. Perhaps the greatest number of cases reported where gall-stones have been obtained has been due to olive oil; the succinate of the peroxide of iron is an old favorite; lately valerianate of amyl and toluylendiamin have been favorably reported. Personally, the use of the salicylates, dioscorea, and olive oil have served best.

A unique case occurring in the practice of Dr. E. S. Swain, of Smithfield, may be found of interest. This case has been reported and the specimens exhibited to the Louisville Pathological Society. Later the specimens were sent to Dr. William Rodman, of Philadelphia. The use of dioscorea and olive oil had been suggested in this case, which proved to be both biliary calculi and abscess. After several days' exhibition of the two drugs about thirty hollow gall-stones were passed. The supposition is that the lecithin of the olive oil had dissolved out the cholesterine of the calculi, leaving the shell, composed of biliary and lime salts.

In the surgical section of the American Medical Association, in 1900, some few surgeons took the position that a diagnosis of gall-stones was sufficient indication for immediate surgical intervention. However, many surgeons echo the sentiments of A. Chauffard (Year Book of Medicine, 1902), who says "Surgical treatment of hepatic colic is too frequently undertaken before medicinal treatment has been properly tested." Chauffard believes the relief obtained by surgical means is likely to be only temporary, and that the factors which previously caused the formation of calculi still remain present.

The intention of this paper is to urge the use of prophylactic measures in those predisposed by diathesis, sex, age, occupation, by intestinal or hepatic disorders favoring biliary stasis and sepsis and in those previously affected with biliary calculi.

It is not claimed that all cases of cholelithiasis can be cured by medicinal means. It is desired to emphasize the fact that internal medication, either alone or combined with surgical measures, must be instituted before a radical cure can result. In support of this it may be mentioned that only a very small proportion of cases of cholelithiasis ever require surgical intervention, and as mentioned by Chauffard the etiologic factors remain and gall-stones may recur after surgical treatment.

Furthermore, any inflammatory process or any foreign matter in the gall-bladder favors the formation of calculi. A case is reported where a secondary operation showed a portion of a stitch introduced at the first operation formed the nucleus of the subsequent calculus. Fiedler states that symptoms of gall-stones occur after operative procedures in fifteen per cent. of cases. Kelly shows that upon one in every seven cases operated upon by Kehr a second operation was performed. Again, the very occurrence of pain suggests the calculus is small enough to become engaged in the duct, and therefore is likely to be modified by medicinal agents. However, it may be said surgery and medicine are complemental. Neither is independent of the other. Combined, they accord a scientific and perfect treatment to cholelithiasis.—*American Practitioner and News.*

---

### GASTRIC NEURASTHENIA.

BY G. E. HUMPHREY, M.D., CAMBRIDGE SPRINGS, PA.

Neurasthenia is a term used to express a lack of or an exhausted condition of nerve energy. It is divided into three classes, cerebral, gastric and sexual. I ask your kind attention to the second of these, gastric neurasthenia. I know there are many practitioners to whom the terms neurasthenia and gastric neurasthenia do not appeal. They are rather impatient of the discussion of them as treating of something altogether too vague to be considered. But why should it be so? Is not the nervous system a most important one, and should not even its functional derangements receive careful attention with the hope of coming, at some future time, to a better understanding of these so-called neuroses?

Are we not apt to look with pitying and not altogether concealed impatience upon our neurasthenic patients? Too often we feel that our duty is done when we assure them that they have no organic disease and therefore no cause for worry, give them a bromide mixture or other nervine and send them about their business. If we hear, perchance, that they have consulted some other practitioner, we are apt to experience a feeling of chagrin at their lack of confidence, mingled with a sense of relief at being rid of them.

Gastric neurasthenia is a dynamic affection of the stomach in which the gastro-intestinal symptoms and discomfort are unattended with or out of proportion to any chemical changes in the stomach contents; characterized by marked weakness of the nerves which supply the stomach, but by no anatomical lesion of the gastric mucous membrane.

*Etiology.*—It is met with in both sexes and most frequently during young adult life. Constitutional lack of nerve energy. Those races whose nerve force has been drawn on by persecution have a neurotic tendency which predisposes them to neurasthenia, as the Russian Jews. Occupations which require the expenditure of great nerve force and those which are carried on in crowded and illy ventilated rooms. Overstudy, sexual excess, unsatisfied sexual desire and sudden shocks. Given the weakness caused by any of the above and add to that the taking of irritating food and drink and you have the exciting cause furnished.

*Symptoms.*—A feeling of anxiety out of proportion to apparent conditions, loss of flesh and strength, leg weariness and inability to do much walking, the patient at times even being confined to bed for periods of several days; inability to concentrate the mind, often to the extent of the patient being unable to attend to business matters which he could ordinarily easily manage; trivial annoyances are brooded over and the patient is likely to become irritable, vertigo and spots before the eyes may be complained of. Any one of these mental and cerebral symptoms may be the only prominent one. In that case what characterizes it as belonging to this disease is its being associated in time with the digestive period.

There is a feeling of goneness which may come on soon after eating or later on. An accumulation of gas is often complained of with which may be associated the feeling of anxiety above mentioned and which an expulsion of gas relieves. Ewald relates a case of a man whose anxiety at such times became so great that he feared death, the fear disappearing with the expulsion of gas.

We may also have associated with it vascular symptoms, such as tachycardia, at times the pulse becoming irregular and intermittent as well as extremely rapid. Hot flashes are also sometimes complained of. Heartburn not caused by hyperacidity. Tenderness over nerve plexuses. This tenderness may be over the hypogastric, celiac or aortic plexuses and are called Burkhardt's painful points. From these points they radiate toward the epigastrium.

*Differential Diagnosis.*—It is necessary to examine the stomach contents to exclude organic disease of the stomach. The intestines should be thoroughly examined to exclude colitis and retained fecal matter. The whole body should be examined to see whether the symptoms can be accounted for by organic disease outside of the intestinal tract. Henchel says, in the *Edinboro Medical Journal*, January, 1902, quoted in the *Therapeutic Gazette*, April, 1902:

"In any case the first thing to do is to make sure that the neurasthenia is not dependent on the absorption into the system of toxins from the alimentary tract. To this end carefully examine the mouth for the presence of necrosing stumps and for pyorrhea alveolaris."

The genital organs should be examined. The urine should also be analyzed for sugar, albumin and uric acid. The condition of the blood should be examined for anemia and malaria. By such careful examination we eliminate the possibility of the symptom-group being reflex due to diseased condition of some of the above organs or an unhealthy condition of the secretions.

*From Chronic Asthenic Gastritis.*—When carefully studied the apparent similarity of these two diseases disappears. An examination of the stomach contents shows the test meal in asthenic gastritis to be little acted on. There is a large quantity of mucus, the enzymes are diminished, and the hydrochloric acid at times is entirely wanting, whereas in neurasthenia the contents are normal, or nearly so. Diet in the former, when properly regulated, has a beneficial effect, in the latter much less so. The tender points are not present in asthenic gastritis.

*From Ulcer.*—Differentiation between these two is not always possible, especially where ulcer is unattended with severe pain, hemorrhage or vomiting. We must at times await developments. General neurasthenic symptoms point rather to neurasthenia. Superacidity in ulcer is usually very pronounced, while in neurasthenia it may be absent. The tender points of ulcer are more localized, being confined to the epigastrium and back, while those of neurasthenia are found over other points.

If a treatment of rest in bed, a milk diet and alkalies fails to afford relief to the discomfort attending the digestion the probabilities favor its being neurasthenia. Gastroptosis may be mistaken for neurasthenia, but an examination of the stomach and finding it displaced clears up the diagnosis. Myasthenia may also be mistaken for neurasthenia, but in the former are signs of retention of food and, if it has been going on any length of time, there are changes in the stomach contents. The tender points and cerebral symptoms are absent.

*Prognosis.*—It is not fatal. Its duration depends largely upon the ability of the patient to take advantage of general and special treatment. It may last a short time or it may last years.

*Treatment*—1. Treatment of the general neurasthenic conditions. Everything should be made use of which will tend to preserve and increase the nervous energy of the individual.

Collins says: " A long, well-filled purse is a powerful factor in the treatment of neurasthenia." When you take into consideration the expense attending the general measures required in best treating such cases, and when we are obliged to treat those who cannot afford to take advantage of such, we readily realize the force of this remark. But I will first mention an aid to treatment which cannot be measured by its money value. I will quote from an editorial entitled "Psychic Aid in Neurasthenia," in the *New York Medical Record* of November 21st, 1891: "As the sensitive are never sensible, it is well to bear in mind their need, when overthrown, of help from others; help having for its chief ingredients earnestness, sympathy, firmness and good judgment. Sympathy need not necessarily be expressed in words; earnestness can be gentle, firmness may be cheerful and smiling and good judgment quiet and unobtrusive. Unless some one places such qualities at the disposal of the neurasthenic and allows him to construct from them a moral support, the value of strychnine, bromides, baths, electricity, and even small doses of opium is greatly diminished and medical labor more or less in vain.

" It is useless to hope for psychic aid from the family except in rare instances. They are alarmed or irritated, and always unwise. It is the physician alone who can come to the rescue."

Under the head of general treatment may be mentioned rest and change, electricity and hydrotherapy. A sojourn for a few weeks or months at a watering-place or at the seashore or mountains will often work wonders. If the circumstances are such that the patient cannot leave home, the aid of those upon whom the patient depends must be relied on.

Their sympathetic assistance may do much in helping out in the treatment.

Diet should be such as is not too stimulating; milk, fat, white meats, green vegetables, soft, juicy and not over acid or over sweet fruits. If the diet gives comfort it is correct; if not, it should be modified to suit the case. A valuable local remedy is the intragastric douche given at night before retiring or in the morning before breakfast. The temperature of the douche should be regulated according to the irritability of the stomach and the weather. Besides plain water a 1-2000 solution of nitrate silver may be used with advantage. Tonics may be indicated such as iron and strychnine. Nervines may be necessary to quiet the nervousness, as may also hypnotics if other means fail to produce sleep. If the bowels are constipated, massage, oil or glycerine injections and electricity may be used.—*Pennsylvania Medical Journal.*

---

### DOCTORS' FEES.

There is something senseless about doctors' fees to which we should like to call attention. This consists in the practice of attempting to make all fees of the average general practitioner uniform, regardless of the length of time they have been in practice. This is contrary to common sense and usage from time immemorial in other relations of life, and contrary to the so-called law of supply and demand. All other things being equal, there is manifestly a vast difference between the value of the judgment of the doctor fresh from college and that of the one with ten, twenty, or thirty years' experience as a practicing physician behind him. In our estimate we are not including specialists or highly successful general practitioners of medicine who receive relatively fancy prices, but only the bulk of the profession, composed of plodders, hard-workers. Many a beginner, in fact, most of them, stand in their own light by insisting upon receiving the same compensation yielded to practitioners of experience. Much of this ill-judged stand by the younger members of the profession is due to the advice to that effect from older doctors. The judgment of the recent graduate being not nearly so good as that of the practitioner having years of experience, it is manifestly absurd for him to exact the same fee as does the latter. This is all the more so in view of the fact that most general practitioners, whose judgment in matters medical has become ripened by experience, lack as much sense in business matters as they ever did in their entire professional careers. We have no sympathy for the doctor who works incessantly, day and night, for good and bad and no-pay patients alike, denying himself to his family and friends, and foregoing the rest and recreation which his knowledge of physiology tells him that he requires, and which his busy life really makes possible because of the income it brings to him. If the older doctor, who has more work than his health will safely permit him to do, or that a proper regard for the rights of his family will allow, insists upon this incessant grind in the selfish fear that some other doctor may get a few of his patients, he is not worthy of being considered by the beginner in practice, to whom the older man is not willing to leave anything. The younger man, under these circumstances, if he has any business sense, is obliged to see people for whatever he can get out of them, meanwhile and thereby establishing his true value as a physician that will enable him, when he becomes sufficiently busy, to do what the older doctor should have done before him, to wit: Weed out his no-pay, his bad-pay, and then his slow-pay, and, finally, his small-pay patients, as he gradually replaces them by others that are surer and better pay, leaving those which he permits to drop for other beginners to make their living and establish their reputations by.

We contend that no physician should work more than a certain number of hours out of each twenty-four. His office hours should be so arranged, according to the kind of practice he does and the community from which he draws his patients, that his fixed time for having them in his office is reduced to a minimum, with a due regard to the convenience of his patients as well as himself. His outside work should be limited to certain hours as far as this is practicable. The balance of the time he should devote to reading, recreation and rest. As soon as he has sufficient income from his practice to enable him to live comfortably, and yet lay aside something for the future, he should cease his night-work. When once arrived at this stage, he should have at his elbow a younger man, quite fresh from college, to attend to such of his patients as are no longer profitable to him, or who demand attention outside of the hours that he has finally selected within which to practice his profession in or outside of his office. Every man in general practice, who is sufficiently successful to make a fair living, should set himself a time limit of so many hours out of every twenty-four, during which he does not practice his profession except upon rare occasions, and for the best of reasons, preferring to let some one else do the work at this time. He can thus begin the weeding-out process of his less desirable patients, dropping one after another as he finds this time encroached upon, and retaining only those who are the more valuable. In this way he keeps his work within safe limits as regards his health, enabling him to devote a proper amount of time to study and to his family and friends. It likewise assures him, that much sooner, a more desirable and better-paying class of practice.

It is the men who have not the moral courage to do this, who lack the intelligence to see its advisability, that plug along throughout their lives until they are old and infirm, working day and night at everybody's beck and call, until the final call that takes them off the face of the earth, and, in all probability, leave their families more or less destitute. These men are often called good-hearted, generous and philanthropic practitioners of medicine, but they are not so much that as they are unwise, foolish, and lacking in common sense. Many of them are so because they lack the moral courage to refuse. Some are too greedy to give the younger men a chance.—*Medical Council.*

---

### THE CHOICE OF CLIMATE FOR PULMONARY INVALIDS.

The question of a choice of climate for those suffering from pulmonary troubles is one the physician is often called upon to settle. Unfortunately the majority of those so afflicted are unable to leave home at all, but to those more fortunate this is often the only means of saving their lives. The open-air treatment of pulmonary affections which has of late been so strenuously advocated by the profession makes the proper choice of climate a thing of great importance and one to be carefully considered. Many sanitaria for this method of treatment have been built during the past few years all over the country; so that at present the physician has the assurance that whatever part of the country he may select as the proper one to send his pulmonary cases to, they will find comfortable quarters and the care and attention which to this class of invalids is so necessary.

The following indications have been laid down by Sir Herman Weber, of London, a leading authority on the question of altitudes as to the choice of a climate for pulmonary invalids:

In cases with limited disease at one or both apices, without or with only a slight amount of fever, nearly all climates can be made use of, especially great altitudes and sea voyages, if the constitution is a strong one.

Cases with limited local disease and high fever must be at first treated in their homes or immediate neighborhood.

In the majority of cases with extensive disease of one or both lungs, without fever or with only slight fever, treatment at only a moderate elevation, or at warm seaside localities, deserves the preference.

In advanced cases with fever, neighboring sheltered health resorts, with careful supervision, should be recommended.

In cases of progressive tuberculosis, with scattered foci in both lungs and much fever, localities near home, or the home itself, are the best places.

In cases of chronic, slowly progressive phthisis, better results are obtained from warm winter resorts, or sometimes from sea voyages.

Quiescent cases, with extensive damage or cicatrization, are generally better off at only slight elevations.

Cases with albuminuria, without fever, should avoid great altitudes.

The complication of moderate diabetes does not exclude great altitudes, but the latter are injurious in cases of advanced diabetes and emaciation.

Chronic cases, with much catarrh, require places with as little wind as possible.

Great altitudes are contraindicated in chronic cases with extensive emphysema.

For the prevention of scrofula and tuberculosis all healthful climates can be used, but great altitudes have advantages against tuberculosis, and marine climates (including sea voyages) more against scrofula.

The cure of tuberculosis during the early stages is possible in all climates, but climate itself, without careful medical supervision, is generally insufficient. The patient's blind reliance on the climate often leads to errors, to aggravation of the disease, and to death.—*Medical Age.*

---

### PROFESSIONAL HARMONY.

In many of our small towns, as well as in the large cities, throughout this and other States, there is a strange lack of good fellowship and harmony among medical men.

A young man, perhaps a recent hospital graduate, well equipped and with a pleasing manner and an air of confidence in his own ability, acquired by contact and experience, settles in a community where one or two men have practiced medicine for years. In some places he is welcomed by his confrères, and admitted into their confidence after an interview or ripened acquaintance. Harmony is at once established, and the nucleus of a medical society is formed or a lifelong friendship established. The people recognize the new blood of enthusiasm that permeates the newcomer and stimulates the older practitioner. A hospital is suggested, instituted and supported; and the doctor becomes a greater power, his influence broadens, and matters medical are better understood and appreciated. In an adjoining town the newcomer is received coldly, treated discourteously, and his progress hampered. If he is a capable man, attends strictly to his own affairs, and waits for business in an ethical way, keeping out of factional fights, he may win his way into good business. If he is resentful, spiteful, and given to criticism of his untried brethren, discord prevails, and the community is the sufferer. Each physician is soon at sword's point with his fellow, refuses to consult with or assist him, and each tries to outdo the other in vindictiveness and abuse. The result is more suffering of the people, dissatisfaction and distrust, and in the end neglect and decline in sanitary progress in the community.

The harmony or discord, which is so frequent in small towns, is enacted with greater force in the larger cities, and the result is the formation of cliques or factions, which are constantly at war with each other, bending every effort to embarrass new departures, and opposing professional advancement or plans for the good of the people. If a man occupies a State or city appointment, he must fight his way through opposition of men who should support his endeavors. He must be successful in many battles before he is recognized as earnest and honest in his intentions, and when he at last becomes useful to the public and has the support of his colleagues, he is dropped to make way for a new and untried appointee. When a medical man in good standing occupies an important post he should be supported by all right-minded medical men, even if he is not perfect, or does not always agree with everyone else, or is man enough to have ideas of his own and the force and energy to attempt to carry them out. Because of his earnestness, vigor, and push, and the publicity he acquires, he should not be called a politician; rather should he be supported loyally by his associates who, by sinking their individual likes or dislikes, create a feeling of confidence and trust about him for the good of the people.

The large medical societies of large cities are doing much to promote harmony in the medical profession. Harmony among men promotes and makes organization of the profession efficient, powerful, and useful. If the carping critics, fault-finding and jealous, would spend the same amount of time in the study of clinical medicine that they devote to slandering their fellow-workmen the medical profession would soon be so harmonious that it would but ask to cheerfully receive from the public all it is entitled to.

All this is solely to promote a better feeling in the profession, and is not written in the spirit of pique, but rather as a hope of smoothing over what may be an imaginary break in the line of harmony. Let us look on the better side of things, get closer together, cultivate one another, and then stick together in one large mass, and work for the good of all.—*Northwestern Lancet.*

---

### ARSENIC IN SKIN DISEASES.

The value of arsenic in diseases of the skin depends entirely upon an exact knowledge of its therapeutic action and its proper employment to meet specific indications. The former is a qualification that belongs to the science of medicine, and the latter an attribute which relates to the art of medicine. To be a successful physician a man must not only know how to do a thing, but he must do it. Science is necessary, but its proper application is equally important in all the affairs of life.

There are three classes of physicians, who differ radically in their views of arsenic as a remedy in diseases of the skin. One of these classes believe, without knowing why, that it is good for all cutaneous disorders and prescribe it indiscriminately. Another class discard it as useless or harmful under any circumstances. The other class, which is numerically small, understand its therapeutic powers and also its limitations in the cure of disease. This class use it judiciously and are successful physicians.

Now let us see what the claims of arsenic as a specific in skin diseases rest upon. It is known to us as a tonic, as a corrective of malassimilation, as a stimulant of the nervous system, and as a stimulant of the skin and mucous membrane. It is an agent of wonderful power and at the same time, when properly administered, is as safe and harmless as any of the drugs in daily use. The best preparation for ordinary purposes is Fowler's solution, and the standard dose is five minims, to be taken after a meal. This dose may be given continuously for months or years, if necessary, without any injurious results, provided no idiosyncrasy contraindicates its use. It may also be given to infants in proportionally larger doses than any other powerful drug. An infant at the breast will tolerate two minims, and a child from two to six years will take three minims, without injurious effects. When unfavorable effects follow its use the symptoms produced will be uneasy feelings of weight or sinking at the epigastrium, nausea, griping, diarrhea, vomiting, suppression of urine, heaviness of the eyes and congestion of the conjunctiva. Nervous symptoms will be restlessness, sleeplessness, faintness, tingling of the hands and feet, and headache. When such symptoms occur it is only necessary to suspend the medicine for a few days or reduce the dose.

Now the question arises, to what class of cases is arsenic applicable? We know that malassimilation is the fundamental factor in cutaneous diseases. We have just stated that arsenic is a potent corrective of that condition.

We must remember that arsenic exhibits two modes of action: primary or tonic action, by which it regulates assimilation; and secondarily, stimulant action directed to the skin. We conclude then in all chronic inveterate forms of skin disease it may be used to great advantage, while in some cases it will undoubtedly act as a specific. It should also be remembered that acute cases can frequently be converted into chronic forms in a few days, when arsenic can be employed just the same as in the ordinary chronic types. I am convinced from long experience that arsenic, judiciously employed, possesses more power over cutaneous diseases than any other single remedy.—*Medical Era.*

---

### ACUTE GASTRO-ENTERITIS OR SUMMER DIARRHEA OF INFANTS.*

Margaret T. Shutt, Springfield, Ill., gives her treatment as follows:

When an attack has developed the indications are these: to clear out of the alimentary canal all of the offending material present as quickly as possible; to allay vomiting; to administer a food that will not afford a suitable culture-medium for germs; if possible, to destroy germs present, and to maintain the strength if there is much depression. If the child vomits repeatedly, washing out the stomach a single time will usually stop it; if not, minute doses of calomel will allay the gastric irritation in many cases, besides clearing out the intestinal tract, and acting as a powerful intestinal antiseptic.

*Jour. A. M. A., August 2, 1902.

In all cases where the stools are small and offensive, flushing out the colon is of great value, and even if it is only possible to do it a single time it is quite worth while. As a rule, children do not object very much to the high enema. Either a weak boric acid or normal salt solution may be used, and the quantity large enough to ensure its returning clear. The temperature of the solution must be determined by that of the child. If the child is very hot the water used must be cool, while if the child is cold and collapsed a hot salt-water enema will act as a powerful stimulant. Often much undigested food and foul-smelling fecal matter is washed out. If there is no nausea castor oil should be given at once, to clear out the rest of the canal. If there is nausea the calomel alone in 1-10-grain doses, repeated hourly until the characteristic odorless calomel stools appear, must be depended upon. The child must be taken off milk at once, whether it be mother's milk or only an imitation of it, and it must not return to a milk diet until the stools become normal. For the first twelve hours or more it will do better without any food, giving it only cold water that has been boiled. Later a suitable substitute must be furnished. Animal broths, egg-water rice-water, and barley-water (either plain or dextrinized) have all been used, and the writer prefers one of the liquid preparations of beef peptonoids now put up by so many drug houses. This affords a certain limited amount of nourishment in a form that is very easily assimilated and also a very little alcoholic stimulation. Children always take it readily. In addition, the child is always to be given all the cool boiled water that it will drink. When the fever continues high the child must be frequently sponged with cool water. After the bowels are thoroughly cleared out give bismuth subnitrate in 10-grain doses, every two hours until the stools become black or dark green. Usually this treatment is sufficient to relieve an attack, but occasionally after the stools become normal in quality and the other symptoms disappear, the diarrhea continues, due only to an increased peristalsis. In such cases, and in such cases only, she gives opium with great caution, either in the form of one-fourth grain of Dover's powder or a few drops of paregoric. While this treatment is being carried on a change of air works wonders.

